# Benchmarking Molecular Mutation Operators for Evolutionary Drug Design

**DOI:** 10.3390/ijms262311685

**Published:** 2025-12-02

**Authors:** Raúl Acosta Murillo, Patricio Adrián Zapata-Morin, José Carlos Ortiz-Bayliss

**Affiliations:** 1 Department of Microbiology and Immunology, School of Biological Sciences, Universidad Autónoma de Nuevo León, Pedro de Alba SN, San Nicolás de los Garza 66455, Nuevo Leon, Mexico; raul.acostaml@uanl.edu.mx; 2 Tecnologico de Monterrey, School of Engineering and Sciences, Eugenio Garza Sada 2501 Sur, Monterrey 64700, Nuevo Leon, Mexico

**Keywords:** molecule mutation, molecule recombination, computer-aided drug design, genetic operators

## Abstract

This study investigates and compares different molecular mutation strategies to optimize their application as genetic algorithm operators in drug design. We evaluated five distinct mutation methods—Graph-Based Genetic Algorithm, Graph-Based Generative Model, SmilesClickChem, SELFIES Token, and SMILES Token Mutation—by assessing their computational efficiency, validity, and impact on molecular complexity and structural conservation. Our results reveal that the Graph-Based Genetic Algorithm achieves the highest molecular validity (96.5%) while maintaining computational efficiency, making it ideal for rapid iterative drug discovery. SmilesClickChem and Graph-Based Generative Model tend to increase molecular complexity, whereas SF-T simplifies molecular structures, suggesting different applications in lead optimization. Additionally, we analyzed mutation-induced changes in pIC50 potency and found that SELFIES Token caused the most substantial shifts in bioactivity, particularly in SRC-targeted molecules. These findings underscore the importance of selecting the appropriate mutation strategy to balance validity, structural diversity, and computational cost in AI-driven drug design. Our insights help refine evolutionary algorithms for molecular generation and optimize candidate selection in early-stage drug discovery.

## 1. Introduction

Drug design is a crucial aspect of pharmaceutical research aimed at developing new therapeutic agents. The traditional process of drug discovery is highly time-consuming, often taking between 10 and 15 years and costing billions of dollars. Despite these investments, the success rate of drugs reaching the market is relatively low, with only about 13% of candidates passing clinical trials [1].

Drug development fails primarily due to four key factors. The most common reason, accounting for 40 to 50% of failures, is the lack of clinical efficacy: a drug does not produce the expected therapeutic benefits in human trials despite promising preclinical results. This issue often arises due to discrepancies between animal models, in vitro studies, and human physiology, making it challenging to validate drug targets effectively. Another significant factor, responsible for 30% of failures, is unmanageable toxicity, where drugs that appear safe in early studies later exhibit severe side effects in humans, often due to differences in metabolism, tissue accumulation, or unexpected off-target effects. Furthermore, 10 to 15% of the failures are attributed to poor drug-like properties, including inadequate solubility, permeability, or bioavailability, which prevent effective absorption and distribution in the body. Lastly, around 10% of failures result from a lack of commercial need or poor strategic planning, in which market demand, competition, or company mergers lead to the discontinuation of drug candidates [2].

Computer-aided Drug Design (CADD) primarily operates through two main strategies: Structure-Based Drug Design (SBDD) and Ligand-Based Drug Design (LBDD). SBDD relies on the three-dimensional structure of a target protein, obtained by techniques such as X-ray crystallography or nuclear magnetic resonance (NMR) spectroscopy [3]. This method involves identifying the active binding site of the target protein, followed by molecular docking, which helps predict how a potential drug molecule will fit and bind to the target [4]. Conversely, LBDD is used when the target protein’s structure is unknown. LBDD relies on known molecules (ligands) that bind to the target, utilizing quantitative structure-activity relationships (QSAR) and pharmacophore modeling to predict new drug candidates with similar properties [5].

In drug discovery, Virtual screening (VS) methodologies can be categorized into several algorithmic approaches, including similarity-based, quantitative, machine learning, metaheuristics, and other algorithms [6]. Similarity-based algorithms assume that structurally similar molecules exhibit similar biological activity, using 2D, 3D, quantum-based, and hybrid techniques along with similarity coefficients such as the Tanimoto index to compare molecular structures [7,8,9]. Quantitative algorithms, such as Quantitative Structure-Activity Relationship (QSAR) models, use mathematical equations to correlate molecular properties such as molecular weight, logP, and topological indices with biological activity, aiding in drug optimization [10,11,12].

Machine learning (ML) algorithms, including artificial neural networks (ANNs) [13], support vector machines (SVMs) [13], decision trees (DTs), Bayesian methods [14], and deep learning (DL) [15,16,17] improve hit identification, predict drug–target interactions, and optimize bioactivity DL models. In particular, they have demonstrated success in analyzing large molecular datasets and refining docking predictions.

Meta-heuristics, which are high-level, problem-independent optimization strategies, are designed to efficiently explore large, complex search spaces where exact methods are impractical. They provide a general framework for guiding and combining subordinate heuristics to find good (but not necessarily optimal) solutions within reasonable computational time. Their main strengths include flexibility, adaptability to different problem domains, and the ability to escape local optima to explore globally promising regions of the solution space. Using metaheuristics, we can enhance VS by optimizing search strategies to explore large chemical spaces efficiently. Single-solution approaches, such as Tabu Search (TS) [18] and Simulated Annealing (SA) [19,20], iteratively refine individual solutions. In contrast, population-based methods, including Genetic Algorithms (GA) [21], Differential Evolution (DE) [22], Ant Colony Optimization (ACO) [23], and Particle Swarm Optimization (PSO) [24], improve multiple candidate solutions simultaneously. Each of these methodologies plays a crucial role in improving virtual screening efficiency, reducing drug discovery costs, and accelerating the identification of potential therapeutic compounds.

Among these meta-heuristics, GAs have been widely used in both Structure-Based Drug Design (SBDD) and Ligand-Based Drug Design (LBDD), depending on the evaluation function used. In SBDD, GAs optimize molecular docking by searching for the best ligand conformations and orientations within a protein’s binding site, using a fitness function based on docking scores that evaluate binding affinity [25]. A well-known example is AutoDock (https://autodock.scripps.edu/, accessed on 29 November 2025), which employs a Lamarckian GA to refine affinity within ligand binding poses [26]. In contrast, in LBDD, GAs are used in QSAR modeling to optimize the selection of molecular descriptors, helping to build predictive models that correlate chemical properties with biological activity. Here, the evaluation function assesses how well selected descriptors predict experimental activity, making GAs a key tool in ligand-based virtual screening (LBVS) [27]. Thus, GAs play a crucial role in both drug design strategies, enabling efficient ligand optimization in docking for SBDD and feature selection in QSAR for LBDD.

The success of early-stage drug design depends on generating molecular structures that remain chemically valid and pharmacologically plausible, and that can preserve interactions with their intended targets. Small changes in functional groups, ring systems, or stereochemistry can dramatically alter a ligand’s ability to fit within protein binding pockets, modify its electrostatic complementarity, or disrupt key hydrogen-bonding patterns essential for inhibition. Consequently, mutation operators used in evolutionary algorithms must balance chemical creativity with the preservation of biologically meaningful features. Inefficient or overly disruptive mutations can lead to unrealistic molecules that violate valence rules, form unstable scaffolds, or lose resemblance to drug-like chemotypes, ultimately reducing the likelihood of identifying viable lead compounds.

The main objective of this study is to design and evaluate mutation strategies for generating molecular structures in drug design. Specifically, the study aims to investigate and compare different mutation strategies—string-based, graph-based, and token-based—and identify their strengths and weaknesses across various contexts. Our research focuses on assessing the effectiveness of these operators by analyzing their impact on the quality, diversity, and validity of the molecules they generate. In addition, the study evaluates computational requirements to balance efficiency with performance.

Derived from this work, we can highlight two main contributions:

- We provide a reproducible framework comparing five mutation operators (GB-GA, GB-GM, SCC, SF-T, SM-T) on a common FDA-approved seed set from ZINC20, under a standardized mutation budget (1/3/5) and metrics (validity, runtime, bioactivity conservation, complexity, and diversity).
- We generate an actionable selection guide, in which we translate results into practical recommendations. For example, use GB-GA for fast, high validity local optimization; SCC for reaction-based growth with property shifts; SF-T for rapid, high-diversity exploration; GB-GM for data-consistent (often heavier) chemistries; and reserve SM-T for quick prototypes due to low validity.

### 1.1. Background and Related Work

Molecular property optimization methods encompass four key categories, each grounded in distinct fundamental principles: Bayesian inference-based techniques [28], reinforcement learning frameworks [29], encoder-decoder architecture models [30], and evolutionary algorithms [31]. Building on this taxonomy, the sections that follow examine representative algorithms—MolDQN, MARS, JTVAE, RationaleRL, REINVENT, and evolutionary strategies such as RGA and classical genetic algorithms—to illustrate how these paradigms shape the action space, validity guarantees, data requirements, and multi-objective trade-offs observed in practice.

Zhou et al. introduced MolDQN, which formulates molecular design as a Markov decision process (MDP) and utilizes Deep Q-Learning (DQN) for molecule optimization [29]. MolDQN directly modifies molecules through chemically valid actions, ensuring 100% chemical validity during generation. Unlike many existing models, it eschews pre-training on datasets and instead learns from scratch using multi-objective reinforcement learning. It leverages bootstrapped DQN for deep exploration by training multiple Q-functions on different subsets of samples and applying ϵ-greedy policies to balance exploration and exploitation.

MARS offers a flexible approach to drug discovery by generating molecules through iterative modifications of molecular graph fragments [32]. Using annealed Markov chain Monte Carlo (MCMC) sampling, it efficiently explores chemical space. Graph neural networks (GNNs) guide the selection of candidate modifications, with the proposal network adapting on-the-fly from generated samples, thereby eliminating the need for external annotated data. MARS excels in multi-objective optimization, achieving high success rates, novelty, and diversity across various drug design tasks.

JTVAE introduces a two-phase molecular graph generation process: first, constructing a tree-structured scaffold from chemical substructures, followed by assembly into a complete molecule using graph message passing networks. This method ensures that intermediate structures remain chemically valid, unlike atom-by-atom generation techniques. JTVAE encodes molecules into dual latent representations—one for the tree structure and one for the graph—which are decoded back into molecular graphs. The model demonstrates superior performance in generating high-scoring molecules through Bayesian optimization within its latent space [33].

RationaleRL is tailored for multi-objective molecular generation by assembling molecules from interpretable substructures, known as rationales, which are responsible for specific chemical properties. The model employs Monte Carlo tree search to extract these rationales and uses reinforcement learning to combine them into complete molecules while optimizing for multiple property constraints. This method enhances accuracy, novelty, and diversity of generated compounds while maintaining transparency through interpretable rationale selection [34].

REINVENT is a comprehensive generative AI framework that leverages recurrent neural networks (RNNs) and transformers for molecular design. It incorporates reinforcement learning, transfer learning, and curriculum learning to generate molecules in the form of SMILES strings. Supporting a range of design tasks including de novo design, scaffold hopping, and molecular optimization, REINVENT integrates these capabilities into a single platform, allowing systematic property optimization with extensive customization via user-friendly configuration files [35].

The Reinforced Genetic Algorithm (RGA) combines reinforcement learning with genetic algorithms for structure-based drug design, using neural networks to guide crossover and mutation based on 3D protein-ligand structures. This improves stability, optimization, and knowledge transfer across targets. RGA outperforms baselines in docking scores and robustness, but crossover operations may compromise molecular synthesizability [36]. While RGA outperforms baselines in docking scores and robustness, it relies on pre-trained neural networks that require large protein-ligand complex datasets and significant computational resources for training and inference.

Genetic algorithms (GAs) are heuristic search methods inspired by natural selection and inheritance, where solutions are generated through genetic operators such as selection, mutation, and recombination of the best candidates, determined by an evaluation function [37]. After selecting solutions for reproduction, the algorithm applies crossover and mutation to produce new solutions while preventing the search process from getting trapped in local optima [38]. In this context, the representation of solutions plays a crucial role, as it influences the choice of the genetic operators [39].

GAs have been applied at various stages of drug design and optimization, such as accelerating global search even when there is a high degree of molecular freedom due to different positions, orientations, and conformations [40,41]. GAs have also helped predict atomic or molecular cluster structures [42] and have been used for feature selection to address dimensionality challenges in QSAR [43,44]. In molecular docking, GAs have been employed to design molecules with high binding energy [25], and they are integral to software like GOLD (https://www.ccdc.cam.ac.uk/solutions/software/gold/, accessed on 29 November 2025) and AutoDock (https://autodock.scripps.edu/, accessed on 29 November 2025). In addition, GAs have been used in QSAR studies to design and optimize inhibitors [45,46].

Challenges in molecular structure generation include ensuring that molecules are valid according to their bond and valence rules [45,47], meeting physicochemical filters such as Lipinski’s Rule of Five [48], and achieving novelty or uniqueness [49]. Additional challenges include molecule complexity, which we assess using the Physicochemical Complexity(PCI) [50], evaluating through a custom complexity index relating the number of H+ acceptors and donors, and improving efficacy, as GAs iteratively optimize solutions [51].

In GAs, mutation operators can help maintain population diversity by randomly altering offspring [51], thereby preventing a uniform population from evolving further [52]. In drug design, the choice of the method for generating molecules significantly affects the molecules designed by altering their structure and physicochemical properties [53]. This, in turn, affects the bioactivity of the mutated molecules, highlighting the importance of exploring various mutation methods.

The literature describes several molecular representation languages developed for compatibility with Deep Learning (DL) models. One of the most common is the Simplified Molecular Input Line Entry System (SMILES), which encodes molecules as strings [54]. However, SMILES strings can sometimes fail to correspond to valid molecules, leading to the development of the SELFIES system, which ensures valid molecular representations [55,56]. Molecules can also be represented in binary form using fingerprints, such as MACCS keys, which depict a molecule as a vector indicating the presence or absence of structural features [57]. Another approach, Extended Connectivity Fingerprints (ECFP4), considers all substructures associated with certain features [58].

When using GAs for drug design, it is common to use ECFP fingerprints (topological fingerprints) to identify substructures and similarity searches. These fingerprints are subject to crossover, mutation, and subsequent decoding into SMILES using Recurrent Neural Networks (RNNs) [59]. Furthermore, Spiegel et al. developed the SMILESClickChem library, which uses the SMART notation and 94 predefined mutation reactions, and SMILESMerge, which identifies and combines the largest shared substructures between parent molecules to generate offspring [25,60,61]. Similarly, Jensen designed a generative model for exploring the chemical space, incrementally adding bonds and atoms to a molecule based on probabilities derived from the ZINC database [62].

For clarification, we provide a summary of these works in Table 1.

### 1.2. A Quick Review of Similar Works on Comparing Algorithms in Molecular Mutations

Fechner and Schneider’s Flux (2) compares several molecular mutation and crossover operators inside a fragment-based evolution strategy for ligand-based de novo design, analyzing how different operator combinations affect diversity and convergence [63]. In their work, all operators act within the same RECAP fragment framework. This work lacks a comparison across fundamentally different representations (graph vs. SMILES vs. SELFIES vs. reaction-based) and does not analyze multidimensional behavior, such as complexity shifts, potency perturbations, and structural diversity, under a unified protocol as you do.

Zhu et al. introduce CALM, a chemical genetic algorithm with a specific molecular matrix representation and custom crossover/mutation operators, and show that their GA outperforms several deep generative baselines on property-optimization tasks [64]. The authors focused on proposing a new GA framework and demonstrating its performance, rather than systematically benchmarking multiple, heterogeneous mutation operators. This way, mutation is treated as a component of a single algorithm rather than the primary object of study.

Prentis et al. present DOCK_GA, a 3D genetic algorithm that evolves ligands using fragment-based mutations and crossovers within the DOCK6 environment for structure-based design [65]. They carefully engineer 3D mutation operations, but the work fails to compare different mutation paradigms or representations. Instead, it evaluates a single docking performance metric for each evolution scheme, without operator-level ablation across multiple mutation strategies.

Krenn et al. analyze robustness to random mutations in SMILES, DeepSMILES, and SELFIES string representations, showing that SELFIES maintains 100% validity under random token mutations while SMILES and DeepSMILES quickly break [56]. This is a representation-level comparison under random mutation, not an algorithmic benchmark of mutation operators in an optimization setting, and it does not cover graph-based or reaction-based mutations, nor multi-objective descriptors or potency shifts.

Nigam et al. use SELFIES-based local edits to traverse chemical space and explicitly compare how random mutations in SMILES, DeepSMILES, and SELFIES differ in validity and diversity [66]. Similar to other works, the comparison is confined to string encodings and random/self-guided mutations within that family. No cross-paradigm benchmark includes graph-based and reaction-based operators, nor is there any operator ranking across multiple chemically meaningful dimensions.

More recent evolutionary and fragment-based methods, such as Kawai’s similarity-driven evolutionary de novo design [67], also define specific mutation schemes and sometimes explore parameter settings, but evaluate a single framework per paper. They do not set up a unified benchmark in which multiple, structurally different mutation operators are plugged into the same backbone and compared on validity, complexity, structural change, potency perturbation, and computational cost.

Taken together, these works show either comparisons of operators within one representation, the introduction and evaluation of a single new GA/mutation scheme, or robustness comparisons between string encodings under random mutation. None of the works revised in this section provides the kind of cross-representation, operator-centered benchmark that our study offers. Actually, to the best of the authors’ knowledge, our study is the first to systematically benchmark molecular mutation operators across multiple representation paradigms (graph-based, reaction-based, SMILES, SELFIES) using a shared experimental protocol and multidimensional evaluation framework, enabling operator-level insights and actionable guidelines that have not been available in any previous work.

## 2. Results

### 2.1. Computational Requirement and Validity

**Figure 1:**
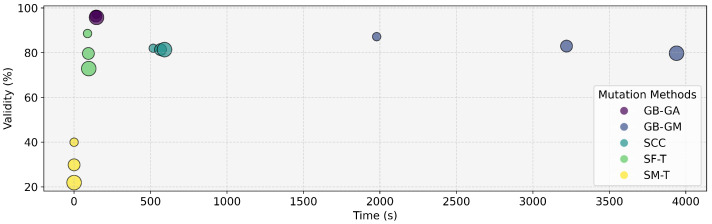
Percentage of validity of the generated molecules and the time it took to generate different mutants per molecule according to GB-GA, GB-GM, SCC, SF-T, and SM-T. The increasing circle size in the visualization corresponds to the number of mutations generated (1, 3, 5).

This study assessed various molecular mutation methods to determine their effectiveness as mutation operators within GAs. We generated various mutants and evaluated their performance based on time, the validity of the generated molecules, and overall efficacy. The results are summarized in Table 2 and Figure 1. The analysis reveals that the GB-GA method achieved the highest validity with an average of 96.5%, followed by GB-GM with 83.3%, SCC with 81.6%, SF-T with 80.4%, and SM-T with 30.6%. This trend regarding the validity is also reflected in the average time taken by each method. Previous research has reported that the SMILES representation only covers a small fraction of the latent space, limiting its ability to fully capture molecular diversity [56].

It is critical to mention that validity was assessed exclusively through an external, uniform post-processing step, not by the internal validity criteria of each mutation/generation operator. After each operator produced a SMILES string, the molecule was subjected to the same RDKit-based sanitization procedure (independent of the operator that generated it). The check consisted of attempting to parse and sanitize the SMILES using RDKit.Chem.MolFromSmiles with full sanitization enabled; molecules that could not be sanitized were labeled invalid. This procedure is implemented in the script validity_check.py in our repository.

Before moving forward, it is relevant to discuss the times reported in Table 2 and Figure 1. We report the average time per molecule to characterize the computational footprint of each operator. However, the practical importance of this parameter depends strongly on the intended application. For small-scale tasks or early hit exploration, differences of a few seconds or minutes per molecule have a limited impact, and the chemical relevance of the generated mutants is typically the dominant factor. However, when these operators are used in large-scale evolutionary pipelines or high-throughput generative workflows involving tens of thousands of mutations (such as within evolutionary computation algorithms), even moderate per-molecule differences can accumulate and affect overall feasibility. For this reason, we include time as part of the benchmark, not as the primary ranking criterion but as a practical indicator of how each operator scales in real use cases.

### 2.2. Conservation of Relevant Features

**Figure 2:**
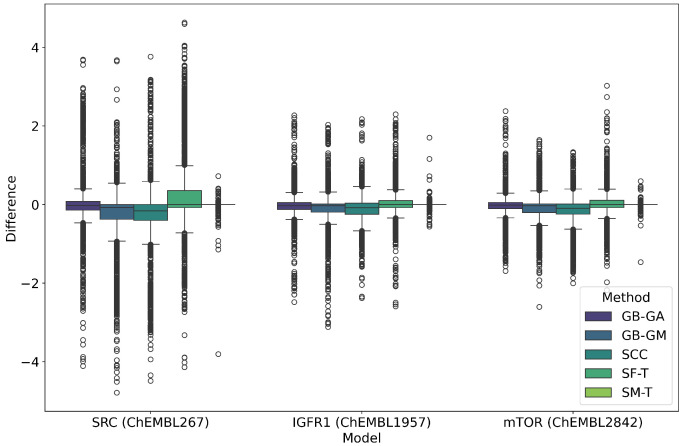
Boxplot of the average differences in pIC50 predicted according to the RF model in the targets SRC, IGF1R, mTOR between the original and mutated molecules according to GB-GA, GB-GM, SCC, SF-T, and SM-T, and (B) distribution of predicted pIC50 values obtained using different methods.

**Figure 3:**
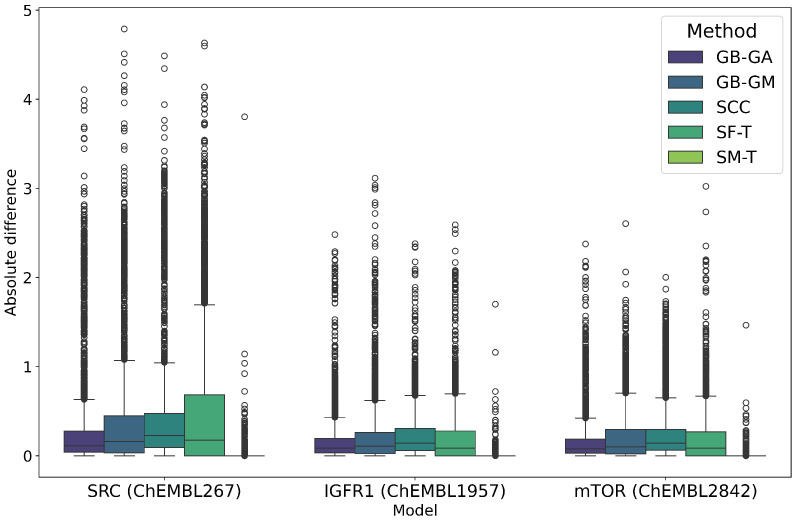
Boxplot of the distribution of predicted pIC50 values for the original and mutated molecules according to the RF model in the targets SRC, IGF1R, mTOR between the original and mutated molecules according to GB-GA, GB-GM, SCC, SF-T, and SM-T.

We evaluated the impact of molecular mutation on the conservation of their relevant features. We generated QSAR models using the random forest algorithm against known therapeutic targets of SRC (ChEMBL267), IGF1R (ChEMBL1957), and mTOR (ChEMBL2842) proteins using preprocessed information from the ChEMBL database. Subsequently, we calculated the pIC50 potency of both the original molecules and the molecules generated by different methods, and we estimated the differences, as shown in Table 3 and Figure 2 and Figure 3.

SRC exhibited the largest average change in predicted pIC50 values at 0.363, followed by IGF1R at 0.166 and mTOR at 0.162. It is important to note that these values represent the average change in absolute terms rather than an increase in pIC50. Among the mutation methods, SF-T resulted in the largest average absolute change (0.613), followed by SCC (0.465), GB-GM (0.418), GB-GA (0.300), and SM-T (0.022). This trend is also reflected in the predicted potency distribution across populations, as illustrated in Figure 2 and Figure 3. The effect of different mutation methods on the predicted bioactivity of molecules, relative to the original compounds across various therapeutic targets, is evident. KL divergence was employed to quantify the differences in pIC50 bioactivity distributions between the original and mutated molecules, as detailed in Table 4, reinforcing the observed trends.

### 2.3. Molecular Complexity

**Figure 4:**
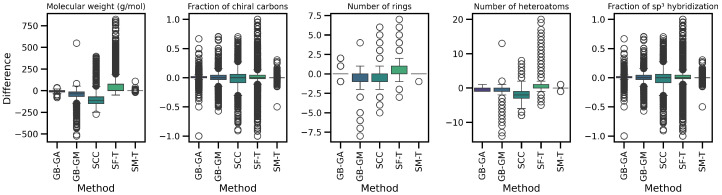
Boxplot of the average differences in complexity descriptors between the original and mutated molecules according to GB-GA, GB-GM, SCC, SF-T, and SM-T.

**Figure 5:**
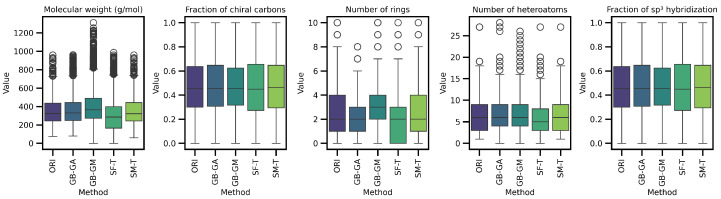
Boxplot of the distribution of the original distribution of complexity descriptors in complexity descriptors between the original and mutated molecule according to different methods.

We assessed the impact of molecular mutation on complexity. Descriptors such as molecular weight, number of rings, number of heteroatoms, fraction of chiral carbons, and fraction of sp^3^ hybridized carbons in both original and mutated molecules were evaluated. The distribution and differences for these descriptors are shown in Table 5 and Figure 4 and Figure 5.

**Figure 6:**
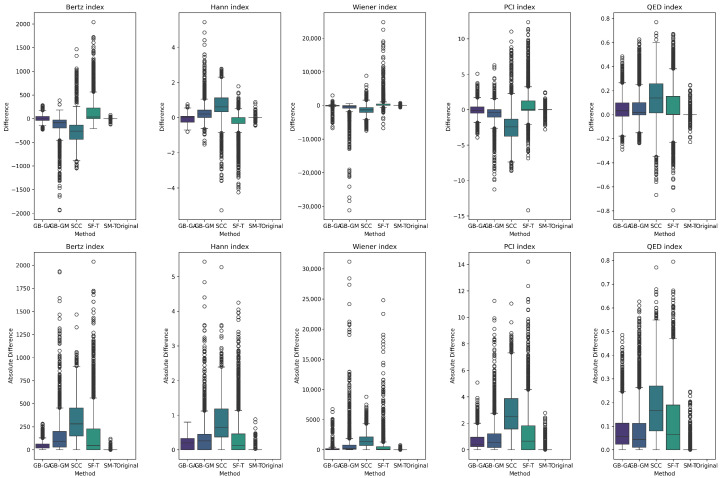
Boxplot of the distribution of the average differences in complexity indices between the original and mutated molecules, and according to GB-GA, GB-GM, SCC, SF-T, and SM-T.

The results reveal a consistent trend across all complexity descriptors, with SCC causing the largest average change (increasing molecular weight, number of rings, and number of heteroatoms), followed by SF-T (decreasing molecular weight and number of rings), GB-GM, GB-GA, and SM-T. Additionally, the effects on complexity indices—such as the Bertz index, Hann index, Wiener index, drug-likeness similarity estimator, and Physicochemical Complexity were analyzed. The results are presented in Table 6 and Figure 6. While the order of change varies depending on the index, the general trend remains consistent for all indices except the Wiener index.

**Figure 7:**
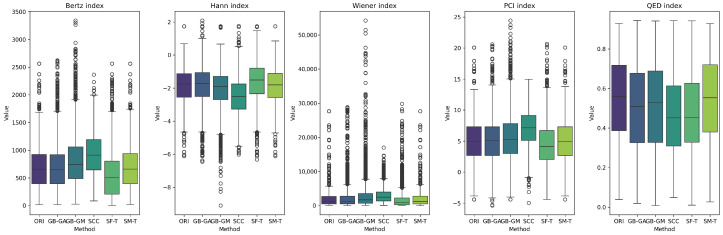
Boxplot of the distribution of the original distribution of complexity indices in complexity indices between the original and mutated molecules, according to GB-GA, GB-GM, SCC, SF-T, and SM-T.

We present the distribution of these descriptors and complexity indices across populations in Figure 4 and Figure 7. These figures show the effects of different mutation methods compared to the original molecules. Notably, the Bertz index and Physicochemical Complexity increase with the SCC method compared to the original molecules. Conversely, there is a reduction in the Hann index, which could indicate a trend toward decreased complexity. This trend is also evident in the KL divergence analysis, which we use to compare the distributions of these properties between the original and mutated molecules. Table 7 demonstrates that the number of rings in molecules mutated through the SCC method exhibited the highest divergence (0.538), reflecting a significant increase in complexity.

### 2.4. Molecular Diversity

**Figure 8:**
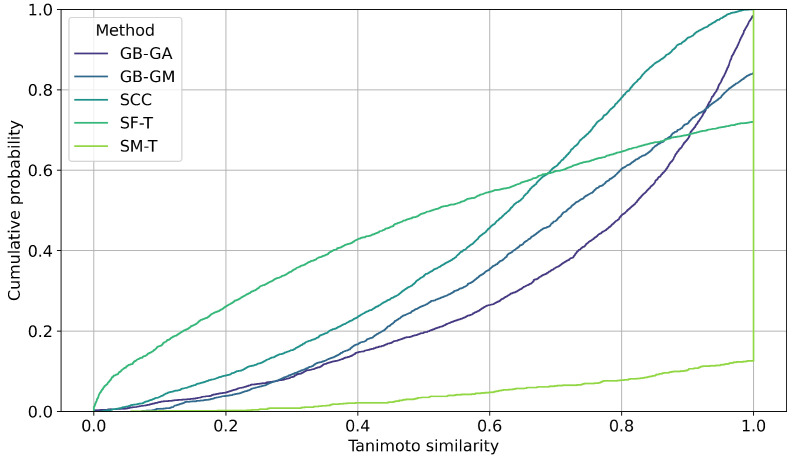
Cumulative distribution of pairwise Tanimoto similarity between the original and mutated molecules generated by GB-GA, GB-GM, SCC, SF-T, and SM-T, calculated using RDKit.

**Figure 9:**
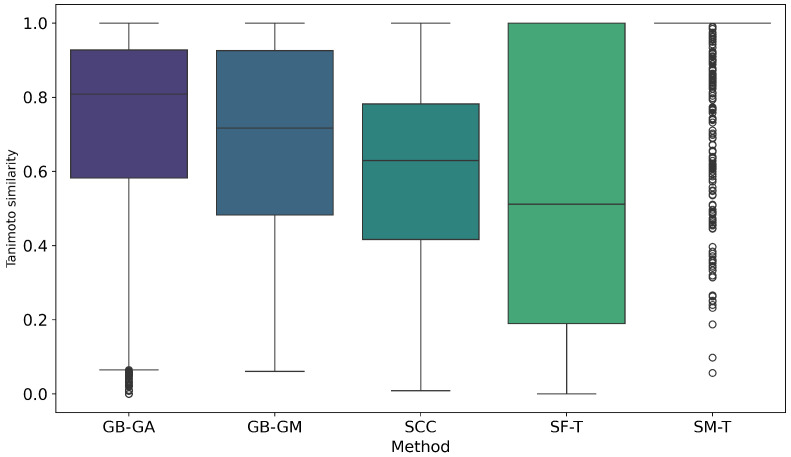
Boxplot of Tanimoto similarity between pairs of original and mutated molecules generated by GB-GA, GB-GM, SCC, SF-T, and SM-T, calculated using RDKit.

We evaluated the impact of molecular mutations on structural similarity using Tanimoto similarity coefficients. The cumulative probability of inter-population similarity (among molecules mutated by a given method) was calculated, as shown in Figure 8. Additionally, we assessed the similarity between the original molecules and the mutated ones. Figure 9 shows the distribution of this similarity. It illustrates how similarity varies depending on the mutation method, with SF-T producing mutated molecules that are significantly different from the originals, while SM-T results in mutated molecules that are nearly identical.

**Figure 10:**
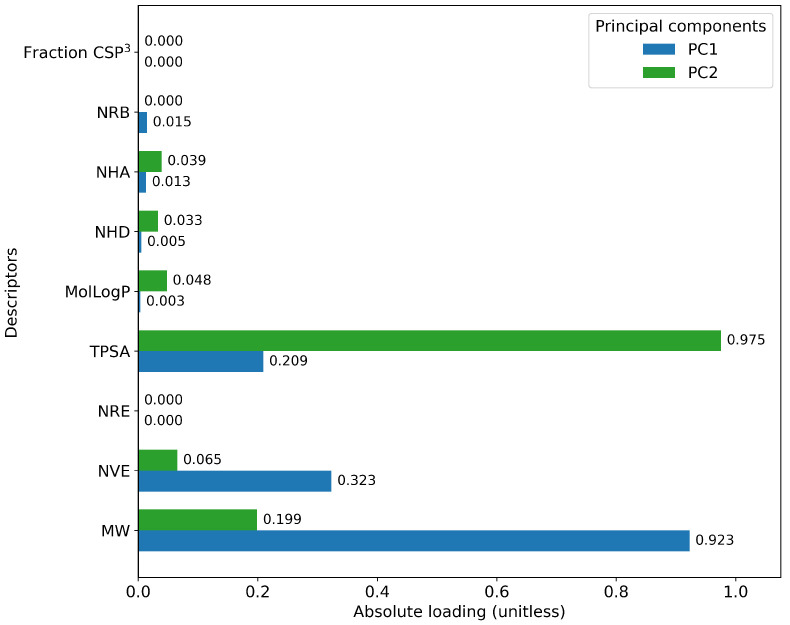
Importance of the molecular descriptors in the first and second components.

**Figure 11:**
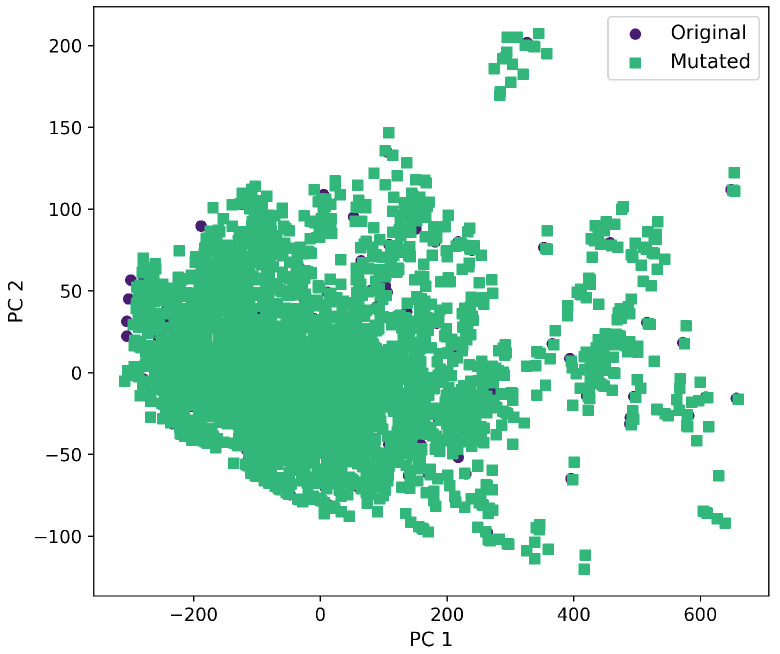
First and second components selected from molecular descriptors using PCA between the original and mutated molecules by the GB-GA method.

**Figure 12:**
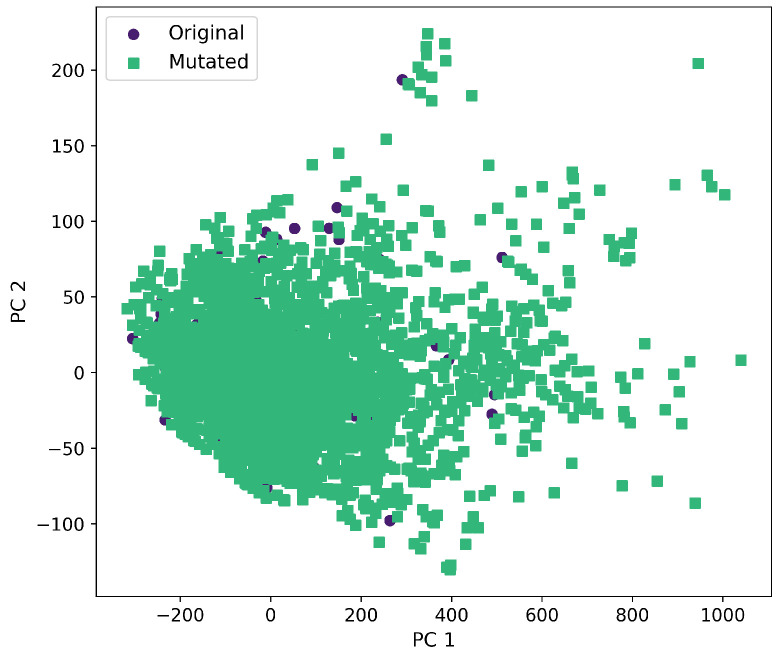
First and second component selected from molecular descriptors using PCA between the original and mutated molecules by the GB-GM method.

**Figure 13:**
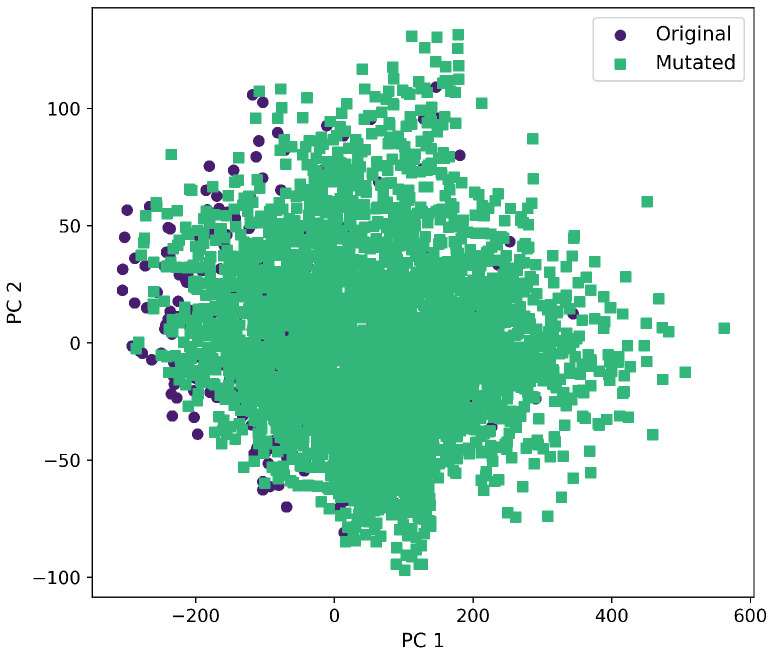
First and second component selected from molecular descriptors using PCA between the original and mutated molecules by the SCC method.

**Figure 14:**
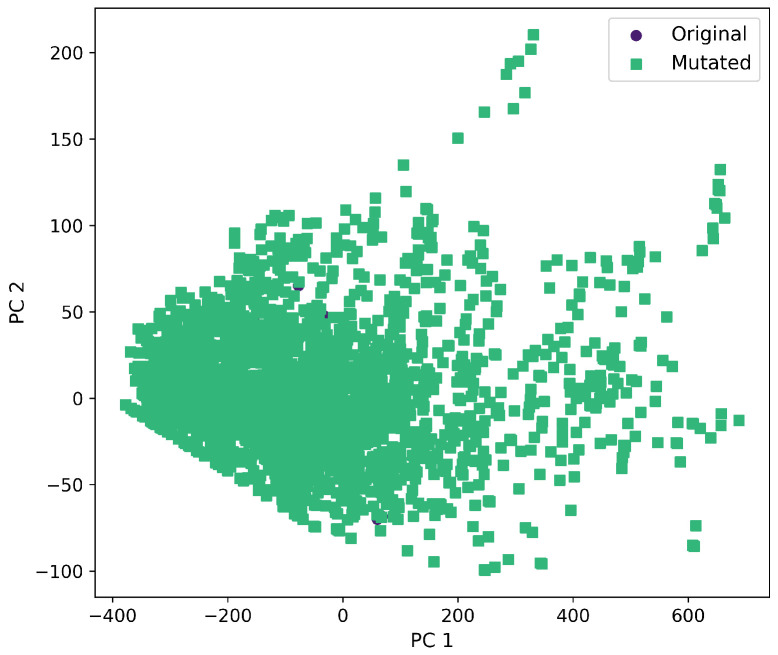
First and second component selected from molecular descriptors using PCA between the original and mutated molecules by the SF-T method.

**Figure 15:**
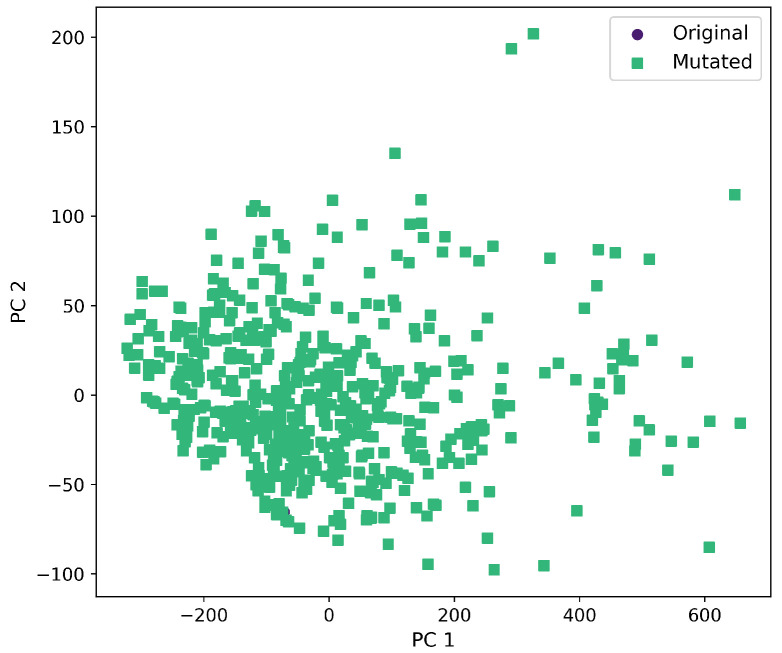
First and second component selected from molecular descriptors using PCA between the original and mutated molecules by the SM-T method.

Furthermore, a comprehensive set of molecular descriptors was calculated, followed by the application of Principal Component Analysis (PCA) to identify the two principal components. Figure 10 shows the significance of each descriptor within these components, where Molecular Weight and TPSA emerged as the most influential. To visualize the distribution of the original and mutated molecules, we generated scatterplots, as shown in Figure 11, Figure 12, Figure 13, Figure 14 and Figure 15. These scatterplots show how the SCC method produced the highest contrast between original and mutated molecules.

Finally, we employed Kullback–Leibler (KL) divergence to compare the distribution of these descriptors between the original and mutated molecules, as illustrated in Table 8. The SCC method exhibited the most significant differences in five out of the seven descriptors measured. For clarity, Table 9 ranks the methods by the magnitude and direction of changes in the characteristics of the mutated molecules.

## 3. Discussion

In this study, we evaluated various molecular mutation methods to characterize their suitability as operators for genetic algorithms. Throughout our experimental setup, we generated several mutants, and we assessed the time taken, validity, and efficacy of the generated molecules. These results are summarized in Table 2 and Figure 1. On average, the GB-GA method exhibited the highest validity at 96.5%, followed by GB-GM (83.3%), SCC (81.6%), SF-T (80.4%), and SM-T (30.6%). In terms of speed, the SM-T method was the fastest (<1s), followed by SF-T (92.4s), GB-GA (144.9s), SCC (558.0s), and GB-GM (3045.7s).

While binary encodings are commonly used in the general approach of mutation operators, drug design requires molecule validity [68]. The GB-GA method’s combination of high validity and relatively low computational time makes it suitable for rapid iterative design. Krenn et al. (2020) [56] reported a 100% validity for MDMA molecule mutation using SELFIES, while DeepSMILES achieved 58.9% and SMILES 26.6%. In this study, the SF-T method had an average validity of 80.4%, similar to the results obtained by Krenn et al., while SM-T exhibited a 30.6% validity. For the SCC (SmilesClickChem) method, Spiegel et al. implemented various molecular filters, resulting in null values if a molecule failed to generate the specified number of mutants [25]. Brown et al. (2019) [53] reported 100% validity with the GB-GM-MCTS method designed by Jensen [62], which aligns closely with the validity observed for GB-GA (96.5%) and GB-GM (83.3%).

We evaluated the impact of molecular mutation on the conservation of relevant characteristics. As mentioned earlier, we developed QSAR models using the Random Forest algorithm for therapeutic targets SRC (ChEMBL267), IGF1R (ChEMBL1957), and mTOR (ChEMBL2842), based on preprocessed data from the ChEMBL database. We estimated the pIC50 potency for both original and mutated molecules, with differences detailed in Table 3, and Figure 2 and Figure 3. On average, the method that most altered the predicted pIC50 potency was SF-T (0.330), followed by SCC (0.314), GB-GM (0.283), GB-GA (0.209), and SM-T (0.016). The QSAR model were the mutated molecules exhibited the greatest changes was SRC (0.363), followed by IGF1R (0.166) and mTOR (0.162).

The distribution of predicted potency is shown in Figure 3, illustrating the effects of different mutation methods compared to the original molecules for various therapeutic targets. We relied on KL divergence to quantify differences in pIC50 potency distributions, with results presented in Table 4. On average, SF-T (0.016) had the greatest impact on the distribution, followed by SCC (0.010), GB-GM (0.009), GB-GA (0.006), and SM-T (0.000). Therefore, the SRC QSAR model showed the most significant changes (0.021) comparing the Original-Mutations molecules, with IGF1R and mTOR each showing changes of 0.002.

Next, we evaluated the impact of molecular mutation on complexity. We analyzed various descriptors, including molecular weight, number of rings, number of heteroatoms, fraction of chiral carbons, and fraction of sp^3^ hybridization carbons for both original and mutated molecules. We present the differences in Table 5 and Figure 5. Additionally, we evaluated complexity indices such as the Bertz index, Hann index, Wiener index, drug-likeness estimator, and Physicochemical Complexity, with results presented in Table 7 and Figure 6. On average, SCC had the greatest impact on molecular weight (113.43), followed by SF-T (64.2), GB-GM (47.6), GB-GA (11.4), and SM-T (0.9). This trend was also observed for the number of rings and heteroatoms, as well as the fraction of chiral carbons and sp^3^ hybridization carbons, reflecting a notable impact on molecular complexity.

We detected a significant increase in complexity for SCC and GB-GM. Spiegel et al. observed that these methods tend to generate heavier molecules, potentially due to the relationship between molecular complexity and interaction, with the Vina scoring function favoring larger molecules [25]. After executing the genetic algorithm, the molecular weight increased by an average of 28%, with a corresponding rise in the number of heteroatoms. In contrast, mutation using SELFIES (SF-T) resulted in reduced molecular complexity by decreasing molecular weight, number of rings, and heteroatoms.

We present the distribution of descriptors and complexity index populations in Figure 5 and Figure 6, reflecting the effect of different mutation methods compared to the original molecules across various therapeutic targets. We compared descriptor distributions between original and mutated molecules using KL divergence, with results detailed in Table 7. SF-T most significantly altered the distributions of molecular weight, number of heteroatoms, and the Bertz index. At the same time, SCC had a greater impact on the number of rings, fraction of chiral carbons, sp^3^ hybridization, and the Wiener and QED indices.

Finally, we estimated the impact of molecular mutation on structure using Tanimoto similarity coefficients. The cumulative probability of inter-population similarity (among molecules mutated by a given method) was calculated, as shown in Figure 8. However, we evaluated the similarity of the original molecules compared to the mutated ones, with results presented in Figure 9. On average, SF-T showed the highest change (0.543), followed by SCC (0.587), GB-GM (0.687), GB-GA (0.724), and at last SM-T (0.957).

Subsequent PCA analysis of molecular descriptors identified two principal components. The importance of each descriptor in these components is illustrated in Figure 10. Scatterplots of original and mutated molecules are shown in Figure 11, Figure 12, Figure 13, Figure 14 and Figure 15, with the TPSA descriptor and molecular weight being those with the highest load in the principal components. The SCC method significantly altered TPSA distribution (0.169), followed by SF-T (0.132), GB-GM (0.058), GB-GA (0.018), and SM-T (0.004). SF-T had the greatest impact on molecular weight (0.094), with SCC (0.044), GB-GM (0.012), GB-GA (0.002), and SM-T (0.000) showing smaller effects. These findings suggest that SCC and SF-T induce substantial changes in molecular structures and highlight the importance of TPSA and molecular weight in evaluating mutation methods. Analyzing these descriptors could optimize the chemical space search by either preserving or altering key molecular features.

Given the results, the GB-GA method stands out due to its high validity (96.5%) and relatively low computational time (144.9s), making it suitable for rapid iterative design processes where the generation of valid molecules is critical. This method also exhibits moderate changes in predicted pIC50 potency and molecular complexity, making it a balanced choice for scenarios where maintaining the integrity of the original molecule’s characteristics is important while also being efficient in terms of computational resources. Therefore, GB-GA could be particularly focused on active hit molecules but could be optimized [69].

On the other hand, the SF-T method is advantageous in scenarios where speed is crucial, being the second fastest (92.4 s) and achieving a relatively high validity rate (80.4%). However, it significantly alters molecular characteristics such as pIC50 potency and molecular complexity, making it more suitable for exploring a wider chemical space and generating more diverse molecular structures [70]. This method might be most appropriate for exploratory phases where structural diversity is prioritized over similarity to the original compounds.

## 4. Materials and Methods

### 4.1. Mutation of Molecules

We will review the following operators among the various mutation methods available in the literature. The initial population will consist of datasets of FDA-approved molecules, extracted from the ZINC20 database [71] (https://zinc.docking.org/, accessed on 30 November 2025). In the following lines, we summarize how each operator works, the representation it acts on, and its typical strengths and limitations.

**Graph-based genetic algorithm (GB-GA)** [62]. GB-GA operates directly on the molecular graph. Primitive edits include adding or removing atoms or bonds, changing atom types or bond orders, and forming/cleaving rings. Each proposed edit is checked against valence and sanitization rules (e.g., with RDKit); invalid offspring are rejected and resampled. This yields high validity and fine control over local changes, at the cost of exploring chemical space more locally around the parent structures.
**Graph-based generative model (GB-GM)** [62]. GB-GM learns data-driven probabilities for graph edits (e.g., which atoms or bonds to add next, given the current context). Molecules are expanded or modified by sampling edits from these learned distributions; some implementations couple this with search (e.g., MCTS) and annealing to bias toward better-scoring candidates. We enforce validity via graph chemistry checks. This approach explores realistic, training-distribution-consistent chemistries, but it can drift toward heavier, more complex products.
**SMILESClickChem (SCC)** [61]. SCC is a reaction-based operator, as it applies curated SMARTS templates (e.g., “click-like” transformations) to a parent and reagents from a building-block library to produce products. Reaction application, atom mapping, and sanitization ensure chemically sensible outputs; rule filters can enforce property bounds (e.g., Lipinski). SCC often increases molecular weight and heteroatom count while enabling chemically interpretable moves.
**SELFIES Token mutation (SF-T)** [56]. SF-T string-level edits (insert/replace/delete) on SELFIES tokens. Because SELFIES encodes valence constraints in the alphabet and grammar, every token sequence decodes to a valid molecule, giving near-100% validity without resampling. Edits can induce larger structural jumps and may bias toward saturated, sp^3^-rich chemotypes unless constrained.
**SMILES Token mutation (SM-T)** [56,72]. SM-T string-level edits on SMILES tokens (insert/replace/delete branches, rings, atom symbols). Fast and simple, but many edited strings are invalid due to unmatched ring indices or parentheses; practical implementations resample up to a cap or validate with RDKit. Variants like DeepSMILES reduce some syntax errors, but validity remains lower than that of SELFIES.

The five operators span complementary design philosophies—graph edits (GB-GA/GB-GM), reaction rules (SCC), and string tokens (SF-T/SM-T)—trading off validity guarantees, magnitude of structural change, and chemical interpretability. This diversity is precisely why we benchmark them side by side in our experiments.

### 4.2. Failure Modes and Operator-Specific Error Sources

Each mutation operator exhibits characteristic failure modes. SMILES Token Mutation (SM-T) frequently produces invalid strings due to unmatched ring indices, incorrect branching syntax, or valence violations, which explains its lower validity scores. In contrast, SELFIES Token Mutation (SF-T) encodes valence rules in its grammar and therefore avoids valence-related errors, although it may generate chemically valid but biologically implausible structures. Graph-based methods (GB-GA and GB-GM) enforce RDKit sanitization; most rejected mutants result from attempted bond additions or ring closures that would exceed atomic valence. SMILESClickChem (SCC) failures occur when reaction SMARTS patterns cannot be matched or when atom-mapping produces non-synthesizable products.

### 4.3. Computational Requirement and Validity

To validate the molecules generated in this work, we will use the percentage of molecules for which RDKit [73] successfully processes the SMILES and generates a molecular object [74], considering bonds and valency [45,47]. Additionally, we will assess the computational requirements of the different operators by measuring the average time taken to generate and process the final population of molecules. Various mutants (one, three, and five) were generated independently, with both time and validity recorded for each scenario. We justify the selection of the number of mutants based on the maximum number of variants observed per compound in the AutoGrow4 method, where five mutations represented the upper limit during the generation process, which ensures that our evaluation aligns with an established method, allowing for a realistic and consistent comparison of computational requirements and validity across different mutation operators [61].

For clarity purposes, the exact alphabets used in our code are SMILES Symbols: FONC()=#12345 and SELFIES Symbols: [epsilon], [Ring1], [Ring2], [Branch1_1], [Branch1_2], [Branch1_3], [F] [O], [=O], [N], [=N], [#N], [C], [=C], [#C]. Regarding the stochastic components, we have documented the hard-coded parameters within our wrapper scripts (selfies_mutation.py and gb_ga.py). While a global seed file was not utilized for the external tools, providing these alphabets and the trained model files ensures the chemical space exploration logic is replicable.

### 4.4. Computational Environment

To provide context for the performance metrics, we executed all our experiments on a standardized computing environment running Ubuntu 22.04.3 LTS (64-bit) allocated with 12 vCPUs (Intel Core i7-12700), 24 GB of DDR4 RAM, and 64 GB of disk storage.

### 4.5. Conservation of Relevant Features

We will assess the preservation of relevant molecular characteristics by evaluating the impact of each mutation operator on the predicted pIC50 values. The pIC50 value, defined as the negative logarithm of the half-maximal inhibitory concentration (IC50), serves as a measure of a compound’s bioactivity in inhibiting its target [75]. Predicted pIC50 values were obtained using Random Forest models, chosen for their high predictive accuracy, minimal hyperparameter tuning requirements, and efficient parallelization capabilities [10]. The QSAR models were trained using protein inhibition data from the ChEMBL database [76], focusing on well-established cancer targets: SRC (ChEMBL267) [77], IGF1R (ChEMBL1957) [78,79], and mTOR (ChEMBL2842) [80,81], all sourced from ChEMBL [76].

To quantify changes in bioactivity, we calculated the absolute differences between the predicted pIC50 values of the original molecules and their mutated counterparts, capturing the magnitude of change without considering directionality. This approach emphasizes the intensity of the change, regardless of whether the bioactivity increases or decreases.

### 4.6. Molecular Complexity

We will assess molecular complexity by considering several factors, including molecular descriptors such as weight, the number of rings and heteroatoms, the fraction of chiral carbons, and the fraction of sp^3^ hybridized carbons [82]. Additionally, we will use Bertz, Hann, and Wiener indices [83], which provide insights into the structural complexity of the molecules. We evaluate the quantitative estimate of drug-likeness using the QED (Quantitative Estimation of Drug-likeness) [84], as well as the Physicochemical Complexity (PCI) [85]. We provide a brief explanation of these descriptors below:

**Number of rings (NR).** NR refers to the count of cyclic structures within a molecule. Rings can influence the molecule’s stability, flexibility, and interactions with biological targets [86,87,88].
**Hann index (HI).** This index is another topological descriptor that quantifies the molecular structure based on its connectivity. It helps in understanding the chemical properties and behaviors of the molecule [89].
**Quantitative Estimate of Drug-likeness (QED).** The QED measures how well a molecule aligns with the characteristics of known drugs, indicating its potential as a drug candidate [84,90].
**Physicochemical Complexity (PCI).** The PCI score is a simple composite physicochemical descriptor combining lipophilicity (logP) with hydrogen-bonding features (HBA–HBD). It is not intended as a rigorous measure of synthetic complexity but may provide a crude proxy for functional-group richness and polarity [91].
**Molecular weight (MW).** MW represents the sum of the atomic weights of all atoms within a molecule. It indicates the size and mass of the molecule, which can affect its pharmacokinetics and pharmacodynamics [92,93].
**Number of heteroatoms (NH).** NH is the count of atoms in a molecule that are not carbon or hydrogen, such as nitrogen, oxygen, or sulfur. Heteroatoms can be crucial in molecular interactions and properties [94].
**Fraction of chiral carbons (QCF).** QCF represents the proportion of carbon atoms in a molecule that are chiral centers. Chiral centers can affect the molecule’s stereochemistry and biological activity [82,95,96,97].
**Fraction of sp^3^ hybridization carbons (HCR).** HCR measures the proportion of carbon atoms in the molecule that are sp^3^ hybridized. Such carbons are typically involved in single bonds and contribute to the molecule’s three-dimensional structure [98,99].
**Wiener index (WI).** WI is a graph-theoretical index that reflects molecular connectivity by summing the distances between all pairs of atoms in a molecule. It provides insight into the molecular shape and size [100,101,102]. The WI is calculated as $\mathrm{WI}=\frac{1}{2}\sum_{i=1}^{n}\sum_{j=1}^{n}d_{ij}$, where *n* is the number of atoms in the molecule and $d_{ij}$ is the shortest path distance between atoms *i* and *j*.
**Bertz index (BI).** BI is a topological index used to describe the complexity of a molecule’s structure, accounting for its connectivity and the arrangement of its atoms [103,104,105,106]. The formula for the BI is given by $\mathrm{BI}=\sum_{i=1}^{n}\log\left(\deg(i)!\right)$, where *n* is the number of atoms in the molecule, deg(i) is the degree of the $i^{\mathrm{th}}$ atom (the number of bonds formed by atom *i*), and $\log\left(\deg(i)!\right)$ is the logarithm of the factorial of the degree of atom *i*. The summation runs over all atoms in the molecule, and the logarithm function is typically taken as the natural logarithm.

### 4.7. Molecular Diversity

As previously mentioned, within GAs, the mutation operators maintain diversity by randomly altering the offspring [51]. Therefore, measuring how much the mutation process truly changes the molecular structure is essential. In order to assess diversity, we rely on Tanimoto similarity coefficients on ECFP4 keys and their cumulative distribution [83]. These calculations will be performed using RDKit [73] and Mordred [107].

In our case, we will determine the following descriptors: Molecular Weight (MW), Number of Valence Electrons (NVE), Number of Radical Electrons (NRE), Total Polar Surface Area (TPSA), LogP (MolLogP), Number of Hydrogen Bond Donors (NHD), Number of Hydrogen Bond Acceptors (NHA), Number of Rotatable Bonds (NRB), and Fraction of sp^3^ Hybridized Carbons (Fraction CSP^3^) computed with RDKit. These descriptors provide insights into various molecular properties influencing drug-like characteristics and complexity [83]. Moreover, we will rely on Principal Component Analysis (PCA) to reduce the dimensionality of the data and identify the two most significant components that capture the majority of the variance [108]. The following lines describe these descriptors.

**Number of Valence Electrons (NVE).** The NVE reflects the total number of electrons in the outermost shell of an atom, which determines how it interacts chemically with other atoms [109].
**Number of Radical Electrons (NRE).** This indicates the number of unpaired electrons in a molecule, which can make it highly reactive [110].
**Number of Hydrogen Bond Donors (NumHDonors).** This counts the number of hydrogen atoms in a molecule that can form hydrogen bonds, which is crucial for molecular interactions in biological systems [111].
**Number of Hydrogen Bond Acceptors (NumHAcceptors).** This counts the number of atoms (such as oxygen or nitrogen) in a molecule that can accept hydrogen bonds, influencing solubility and binding properties [111].
**Number of Rotatable Bonds (NumRotatableBonds).** This is the count of single non-ring bonds, excluding terminal bonds, that can freely rotate, affecting the flexibility and conformational diversity of a molecule [112].
**Total Polar Surface Area (TPSA).** The TPSA measures the surface area of a molecule occupied by polar atoms, typically oxygen and nitrogen, and indicates a molecule’s ability to interact with water (hydrophilicity) [113]. The TPSA is calculated as $\mathrm{TPSA}=\sum_{i=1}^{n}A_{i}$, where *n* is the number of polar atoms in the molecule (typically oxygen and nitrogen) and $A_{i}$ is the polar surface area of atom *i*.
**Octanol-Water Partition Coefficient (LogP).** The LogP is the logarithm of the partition coefficient between *n*-octanol and water, providing an estimate of a molecule’s hydrophobicity or lipophilicity, which affects its ability to cross cell membranes [114,115,116]. The LogP is calculated as $\mathrm{LogP}=\log\left(\frac{\left[C_{\mathrm{Octanol}}\right]}{\left[C_{\mathrm{Water}}\right]}\right)$, where $\left[C_{\mathrm{Octanol}}\right]$ is the concentration of the compound in octanol and $\left[C_{\mathrm{Water}}\right]$ is the concentration of the compound in water.

### 4.8. Distribution Tests

We will conduct distribution tests using the Kullback–Leibler Divergence (KL) technique to evaluate how well the probability distribution *Q* approximates another distribution *P*. This method aims to assess the similarity in the distribution of physicochemical and bioactivity characteristics across different mutation methods [53].

Consistent methodologies were used across different comparisons, including the KL divergence and others employed in this study, to ensure uniformity in the analysis. We will perform the Kullback–Leibler Divergence (KL) technique to compare the distribution of original versus mutated molecules, providing insights into the preservation of molecular characteristics post-mutation. The Kullback–Leibler Divergence is calculated as $D_{\mathrm{KL}}\left(P\;||\;Q\right)=\sum_{x}P\left(x\right)\log\left(\frac{P(x)}{Q(x)}\right)$.

## 5. Conclusions

In conclusion, our study provides a comprehensive evaluation of various molecular mutation methods to assess their suitability as operators for genetic algorithms. We analyzed the generation of mutants, focusing on factors such as time, molecule validity, and overall efficacy. Our results demonstrate that the GB-GA method achieved the highest average validity (96.5%), followed by GB-GM (83.3%), SCC (81.6%), SF-T (80.4%), and SM-T (30.6%). In terms of speed, SM-T proved to be the fastest, while GB-GM was the slowest.

Comparisons with previous research showed consistency in the performance of some methods, such as SELFIES, which achieved 100% validity for MDMA mutation. However, slight variations were noted, such as the influence of SCC’s molecular filters on mutational outcomes. Our study also explored the impact of mutation on preserving molecular characteristics, including the effect on pIC50 potency predictions for therapeutic targets. Notably, SF-T exhibited the greatest average change in predicted potency, with SRC being the most affected target.

Assessment of molecular complexity revealed that methods like SmilesClickChem and GB-GM tended to increase complexity, while SELFIES (SF-T) reduced it by affecting molecular weight and other descriptors. Structural analysis, guided by Tanimoto similarity coefficients, highlighted significant changes induced by the mutation methods, with SF-T and SCC showing the most substantial impacts. Principal Component Analysis (PCA) identified descriptors such as TPSA and molecular weight as key factors in differentiating between original and mutated molecules, underscoring their importance in molecular characterization.

We assessed the impact of molecular mutation on structural changes through Tanimoto similarity coefficients and PCA analysis, showing TPSA and molecular weight as main components. The results highlight that the GB-GA method demonstrates a balance between maintaining molecular integrity and computational efficiency, making it suitable for iterative design processes where both validity and resource management are crucial. Conversely, the SF-T method, while faster and yielding greater structural diversity, significantly alters molecular characteristics, positioning it as a better fit for exploratory phases of drug design.

Overall, our study offers valuable insights into the performance and implications of various molecular mutation methods, highlighting their potential applications in genetic algorithms and molecular design processes. Future research could focus on optimizing these methods for specific applications and exploring their utility across diverse molecular contexts.

Regarding future work, a promising direction is to develop hybrid mutation ensembles that integrate the strengths of multiple strategies. In such an approach, the mutant sets produced by graph-based, reaction-based, SELFIES-based, and SMILES-based operators could be generated in parallel, filtered to remove intersections, and validated using standardized chemical sanitization. The resulting non-redundant, cross-operator mutant pool would combine the high validity and structural fidelity of graph-based edits, the chemically interpretable transformations of reaction-based operators, and the high-diversity jumps introduced by SELFIES mutations. This unified population could provide a richer and more chemically diverse search space for downstream tasks such as docking, QSAR optimization, or multi-objective molecule design. By leveraging the complementary exploration patterns of the individual operators, hybrid ensembles may outperform single-operator pipelines in both early hit expansion and scaffold exploration, offering a practical path toward more comprehensive and adaptive drug development workflows.

## Figures and Tables

**Table 1:** Summary of representative molecular design algorithms, considering their main strengths and shortcomings.

Algorithm	Core Idea	Strengths	Shortcomings
MolDQN	MDP formulation with Deep Q-Learning and chemically valid edit actions.	100% validity, no pretraining, strong multi-objective control.	High computational cost, requires multiple Q-functions, reward-design sensitive.
MARS	Fragment-level edits guided by an adaptive proposal network and annealed MCMC.	High novelty/diversity, no labeled data required.	Sampling can drift, dependent on proposal quality, slow convergence.
JTVAE	Two-stage generation: scaffold via junction tree, then graph assembly.	Valid substructure assembly, strong latent-space optimization.	Complex architecture, requires predefined vocabularies, heavy training.
RationaleRL	RL + MCTS to assemble molecules from interpretable rationales.	Interpretable, good multi-objective balance.	MCTS is expensive, relies on rationale extraction, limited chemical space.
REINVENT	SMILES-based generative RL (RNN/Transformer) with curriculum learning.	Flexible, supports scaffold hopping and optimization.	SMILES syntax errors, diversity collapse risk, chemistry not explicit.
RGA	Neural-network-guided GA operations using protein–ligand structure data.	Improved docking scores, robust exploration.	Requires large structure datasets, expensive, may produce non-synthesizable offspring.
Classical GA	Crossover/mutation on graphs, strings, or fingerprints.	General-purpose, good global search ability.	Validity not guaranteed, heavily representation-dependent.
ECFP-GA	GA on fingerprints, decoding via RNNs.	Useful for similarity/QSAR tasks.	Fingerprint decoding unreliable, structural info lost, validity issues.
SCC	Reaction-based mutations using SMARTS templates.	Chemically interpretable, rule-based transformations.	Tends to increase size/complexity, limited by template library.
GB-GA	Graph edits with chemical sanitization (atom/bond changes, ring edits).	High validity, precise local modifications.	Local bias, slower due to sanitization, limited large-scale edits.
GB-GM	Data-driven probabilities for graph edits learned from chemical datasets.	Realistic, distribution-consistent molecules.	Tends toward heavier molecules, training-data-biased, slow.
SF-T	Token edits on SELFIES with guaranteed validity.	Near-100% validity, simple, large exploratory jumps.	Overly saturated/sp^3^-rich outputs, unstable property changes.
SM-T	Token edits on SMILES (insert/replace/delete).	Very fast, easy to implement.	Low validity, many syntax errors, high resampling needed.

**Table 2:** Time, validity, correctly mutated molecules, and efficiency are assessed for the generation of different numbers of mutants according to various methods. The average across the different mutations is calculated for each method with the best result highlighted in bold.

Method	Mutants	Validity (%)	Molecules	Time (s)	mol/s
GB-GA	1	**97.2**	486	145.2	3.3
	3	96.4	1446	142.4	10.2
	5	95.8	**2396**	147.0	16.3
	Average	96.5	1442.7	144.9	9.9
GB-GM	1	87.2	436	1978.2	0.2
	3	83.0	1245	3219.8	0.4
	5	79.8	1995	3939.1	0.5
	Average	83.3	1225.3	3045.7	0.4
SCC	1	82.0	410	517.6	0.8
	3	81.4	1221	565.0	2.2
	5	81.4	2035	591.4	3.4
	Average	81.6	1222.0	558.0	2.1
SF-T	1	88.6	443	87.7	5.1
	3	79.7	1195	93.3	12.8
	5	72.9	1822	96.2	18.9
	Average	80.4	1153.3	92.4	12.3
SM-T	1	40.0	200	$2.316\times 10^{-3}$	**84,724.9**
	3	29.9	448	$1.986\times 10^{-2}$	22,562.7
	5	21.9	547	$1.098\times 10^{-2}$	49,837.8
	Average	30.6	398.3	$1.106\times 10^{-2}$	52,375.1

**Table 3:** Average difference in the absolute predicted pIC50 value according to the RF model in the targets SRC, IGF1R, and mTOR between the original and mutated molecules, as determined by GB-GA, GB-GM, SCC, SF-T, and SM-T. We have highlighted the largest difference in bold.

Method	Mutants	SRC	IGF1R	mTOR	Average
GB-GA	1	0.298	0.168	0.157	0.208
	3	0.304	0.171	0.152	0.209
	5	0.298	0.170	0.166	0.212
	Average	0.300	0.170	0.158	0.209
GB-GM	1	0.448	0.210	0.217	0.292
	3	0.377	0.227	0.212	0.272
	5	0.428	0.220	0.206	0.285
	Average	0.418	0.219	0.212	0.283
SCC	1	0.466	0.228	**0.249**	0.314
	3	0.476	**0.237**	0.247	0.320
	5	0.452	0.227	0.241	0.307
	Average	0.465	0.231	0.246	0.314
SF-T	1	0.539	0.151	0.135	0.275
	3	0.613	0.210	0.189	0.337
	5	**0.689**	0.230	0.216	**0.378**
	Average	0.613	0.197	0.180	0.330
SM-T	1	0.027	0.015	0.007	0.016
	3	0.014	0.013	0.011	0.012
	5	0.024	0.017	0.016	0.019
	Average	0.022	0.015	0.011	0.016
Average		0.363	0.166	0.162	0.230

**Table 4:** KL divergence values between the distributions of pIC50 bioactivity against different therapeutic targets of the original molecules vs. the mutated molecules according to GB-GA, GB-GM, SCC, SF-T, and SM-T.

Method	SRC	IGF1R	mTOR	Average
GB-GA	0.014	0.002	0.002	0.006
GB-GM	0.021	0.004	0.003	0.009
SCC	0.025	0.003	0.003	0.010
SF-T	0.043	0.003	0.003	0.016
SM-T	0.001	0.000	0.000	0.000
Average	0.021	0.002	0.002	0.008

**Table 5:** Average absolute difference in complexity descriptors between the original and mutated molecules for different mutation methods, as computed with RDKit. We have highlighted the largest differences in bold.

Method	Mutants	MW	NR	NH	QCF	HCF
GB-GA	1	11.023	0.286	0.319	0.048	0.048
	3	11.412	0.273	0.349	0.043	0.043
	5	11.693	0.280	0.341	0.043	0.043
	Average	11.376	0.280	0.337	0.045	0.045
GB-GM	1	49.089	0.516	0.828	0.063	0.063
	3	45.883	0.500	0.841	0.061	0.061
	5	47.850	0.516	0.845	0.062	0.062
	Average	47.607	0.510	0.838	0.062	0.062
SCC	1	111.204	0.824	2.346	0.105	0.105
	3	**114.642**	0.880	2.403	0.106	0.106
	5	114.461	0.843	**2.410**	**0.110**	**0.110**
	Average	113.436	0.849	2.387	0.107	0.107
SF-T	1	64.978	0.648	1.364	0.103	0.103
	3	58.952	0.770	1.165	0.095	0.095
	5	68.837	**0.908**	1.397	0.108	0.108
	Average	64.256	0.775	1.309	0.102	0.102
SM-T	1	0.509	0.000	0.060	0.004	0.004
	3	0.726	0.000	0.056	0.004	0.004
	5	1.435	0.002	0.115	0.008	0.008
	Average	0.890	0.001	0.077	0.005	0.005

**Table 6:** Average absolute difference in complexity indices between the original and mutated molecules for each mutation method (GB-GA, GB-GM, SCC, SF-T, and SM-T), computed with RDKit. The largest differences are highlighted in bold.

Method	Mutants	BI	HI	WI	QED	PCI
GB-GA	1	46.889	0.187	183.570	0.079	0.681
	3	46.659	0.187	169.972	0.080	0.648
	5	47.128	0.185	178.343	0.079	0.666
	Average	46.892	0.186	177.295	0.079	0.665
GB-GM	1	140.972	0.301	923.521	0.081	0.972
	3	139.074	0.320	768.611	0.081	0.908
	5	149.420	0.332	808.420	0.081	0.945
	Average	143.155	0.318	833.517	0.081	0.942
SCC	1	307.516	0.764	1487.393	0.180	**2.841**
	3	**323.713**	**0.833**	**1585.059**	**0.187**	2.789
	5	319.383	0.813	1566.556	0.186	2.792
	Average	316.871	0.803	1546.336	0.184	2.807
SF-T	1	90.544	0.202	332.273	0.073	0.768
	3	153.008	0.335	568.392	0.116	1.212
	5	197.828	0.434	722.413	0.128	1.480
	Average	147.127	0.324	541.026	0.106	1.153
SM-T	1	0.603	0.014	3.995	0.004	0.083
	3	1.340	0.012	7.634	0.006	0.078
	5	2.405	0.028	14.333	0.011	0.153
	Average	1.449	0.018	8.654	0.007	0.104

**Table 7:** KL divergence values between the complexity distributions between the original molecules vs the mutated molecules according to GB-GA, GB-GM, SCC, SF-T, and SM-T. We have highlighted the largest difference in bold.

Methods	MW	NR	NH	QCF	HCF	BI	WI	QED
GB-GA	0.002	0.143	0.007	0.134	0.134	0.006	0.006	0.031
GB-GM	0.013	0.363	0.026	0.235	0.235	0.036	0.084	0.040
SCC	0.044	**0.538**	0.070	**0.374**	**0.374**	0.108	**0.228**	**0.098**
SF-T	**0.124**	0.372	**0.132**	0.314	0.314	**0.173**	0.225	0.091
SM-T	0.000	0.000	0.002	0.002	0.002	0.000	0.000	0.003

**Table 8:** Descriptor values for GB-GA, GB-GM, SCC, SF-T, and SM-T mutation methods. The best values are highlighted in bold.

Descriptor	GB_GA	GB_GM	SCC	SF-T	SM-T
MolWt	0.002	0.012	0.044	**0.094**	0.000
NumValenceElectrons	0.001	0.012	0.043	**0.089**	0.000
TPSA	0.018	0.058	**0.169**	0.132	0.004
NumHDonors	0.347	0.643	**1.209**	0.697	0.123
NumHAcceptors	0.023	0.046	**0.178**	0.134	0.007
NumRotatableBonds	0.302	0.142	**0.649**	0.530	0.012
FractionCSP^3^	0.134	0.234	**0.374**	0.282	0.002

**Table 9:** Order of different aspects of the mutation methods in the headers: (↑): Ascendant order. (↓): Descendant order. For pIC50 values, Com. Dec. (Complexity Descriptors), Com. Ind. (Complexity Indices), and Diversity the order is determined by the absolute difference between the original and mutated molecules, the arrow corresponds if the average difference is positive (increases ↑) or negative (decreases ↓) when mutated.

	Speed ↑	Validity ↓	pIC50 ↓	Com. Dec. ↓	Com. Ind. ↓	Diversity ↓
GB-GA	3	1	4 ↑	4 ↑	4 ↓	4
GB-GM	5	2	3 ↑	3 ↑	3 ↑	3
SCC	4	3	1 ↑	1 ↑	1 ↑	2
SF-T	2	4	3 ↓	2 ↓	2 ↓	1
SM-T	1	5	5 ↑	5 ↑	5 ↑	5

## Data Availability

All data supporting the reported results—including the results with the source code used to train, test, and save the models, are openly available at https://github.com/RollerCoaster1899/MolecularMutation (accessed on 29 November 2025). The repository provides scripts, configuration files, and instructions to reproduce the experiments. Public benchmark datasets used in this study are referenced in the repository with links to their original sources; redistribution follows the original licenses.

## Abbreviations

The following abbreviations are used in this manuscript:

ACO	Ant Colony Optimization
APC	Article Processing Charge
ANN	Artificial Neural Network
BI	Bertz Index
CADD	Computer-Aided Drug Design
ChEMBL	ChEMBL bioactivity database
CSP^3^	Fraction of sp^3^-hybridized carbons (RDKit descriptor)
DE	Differential Evolution
DL	Deep Learning
DQN	Deep Q-Network
DT	Decision Tree
ECFP4	Extended Connectivity Fingerprint (diameter 4)
FDA	U.S. Food and Drug Administration
GA	Genetic Algorithm
GB-GA	Graph-Based Genetic Algorithm
GB-GM	Graph-Based Generative Model
GB-GM-MCTS	Graph-Based Generative Model with Monte Carlo Tree Search
GNN	Graph Neural Network
HI	Hann Index
HCF	Fraction of sp^3^-hybridized carbons (alt. label used in tables)
HCR	Fraction of sp^3^-hybridized carbons
IGF1R	Insulin-like Growth Factor 1 Receptor
JTVAE	Junction Tree Variational Autoencoder
KL	Kullback–Leibler (divergence)
LBVS	Ligand-Based Virtual Screening
LBDD	Ligand-Based Drug Design
LogP	Octanol–Water Partition Coefficient (logarithm)
MACCS	Molecular ACCess System (structural keys)
MAE	Mean Absolute Error
MARS	Markov Molecular Sampling
MCMC	Markov Chain Monte Carlo
MDP	Markov Decision Process
MolLogP	Calculated logP descriptor (RDKit)
mTOR	mechanistic Target of Rapamycin
MW	Molecular Weight
NH	Number of Heteroatoms
NHA	Number of Hydrogen-Bond Acceptors
NHD	Number of Hydrogen-Bond Donors
NMR	Nuclear Magnetic Resonance
NRE	Number of Radical Electrons
NR	Number of Rings
NRB	Number of Rotatable Bonds
NVE	Number of Valence Electrons
PCA	Principal Component Analysis
PCI	Physicochemical Complexity
PSO	Particle Swarm Optimization
QCF	Fraction of Chiral Carbons
QED	Quantitative Estimate of Drug-likeness
QSAR	Quantitative Structure–Activity Relationship
R^2^	Coefficient of Determination
RDKit	RDKit cheminformatics toolkit
REINVENT	Framework for de novo molecular design (RNN/Transformer + RL)
RF	Random Forest
RGA	Reinforced Genetic Algorithm
RMSE	Root Mean Square Error
RNN	Recurrent Neural Network
SA	Simulated Annealing
SCC	SMILESClickChem
SELFIES	Self-Referencing Embedded Strings
SF-T	SELFIES Token mutation
SM-T	SMILES Token mutation
SMILES	Simplified Molecular Input Line Entry System
SMARTS	SMILES Arbitrary Target Specification
SBDD	Structure-Based Drug Design
SRC	Proto-oncogene tyrosine-protein kinase Src
SVM	Support Vector Machine
TPSA	Topological Polar Surface Area
TS	Tabu Search
VAE	Variational Autoencoder
VS	Virtual Screening
WI	Wiener Index
ZINC20	ZINC database (version 20) of purchasable compounds
